# Case of Placenta Prævia

**Published:** 1849-12

**Authors:** Robert Burns

**Affiliations:** Frankford, Pennsylvania


### *Case of Placenta Prævia.* By Robert Burns, M. D., *of Frankford, Pennsylvania.*

Mrs. C. D. C——y, aged 30 years, of spare habit, and delicate but symmetrical proportions, in her eighth pregnancy. During the early months she enjoyed good health and spirits, which met with no interruption until the 26th of September last, when she was seized with flooding to a considerable extent. The circumstance did not produce much alarm, as, in a former pregnancy, she had a similar attack from venous congestion, which was speedily arrested by venesection and quietude. Consequently I was not called in until the following day. By this time the discharge had somewhat moderated, and the acetas plumbi and opium were prescribed, combined with absolute quietude, cold drinks and light aliment. She was at this time about the seventh month of gestation, and *per tactum* the neck of the uterus was found in a normal state, and the os uteri closed. This treatment in a few days had the desired effect, and the patient resumed her wonted duties, being advised to be very careful and avoid over exertion or any other exciting cause. Nothing disturbed this state of things for a period of two weeks, when a more severe recurrence of the hemorrhage took place. Finding some febrile action and sanguineous congestion, I bled her freely, and directed a strict observance of the previous directions, with confinement to the recumbent position. From the commencement of the case I was persuaded it was one of placental presentation, and determined to adhere to this opinion until it should be proved otherwise, lest, by entertaining minor considerations, it should have a fatal termination.

My patient being very intelligent, and anxious to understand her condition, I did not hesitate to explain my fears, in order that she might the more willingly comply with my efforts. Things continued much the same until the evening of the 6th of November, when the moderate discharge which had existed for several days became suddenly increased, although every precautionary direction had been carried out. On examination I found more obliteration of the neck of the uterus, and slight dilatation of the os uteri, although this was scarcely perceptible. I now resolved on the use of the tampon, and proceeded to its application in the following manner: About 7 o'clock, P.M., on the 6th inst., as above stated, without changing the position of my patient, which was that on the left side, I introduced a large, tri-valved speculum uteri, which, together with the os externum, were well lubricated with adeps; the instrument being carried up to its full extent was dilated by the operation of the screw. I then took a piece of firm sponge, sufficiently large to fill the whole vagina to considerable distention: having now fixed the speculum securely with the left hand, I passed the sponge into the speculum by a firm rotary motion, guarding at the same time against too much pressure upon the uterus by an antagonistic force with the left hand. The sponge now lodged within the speculum, I introduced the director of the instrument upon the tampon, and while pressing the sponge forward with the right hand withdrew the instrument in the same steady manner with the left hand, the screw being previously reversed. The tampon now was securely and very effectually applied, having produced by its introduction so little uneasiness to the patient that she scarcely in the least complained. Here I would remark, that in this manner I have operated for the last two years with great satisfaction and facility. Whether the plan is adopted by other obstetricians, or ever has been, I am yet to be informed—certain it is that it has been entirely original with me. A compress *ad vulvam* supported by the T bandage was now firmly applied, and special directions given for the night.

Early in the morning of the 7th inst. my patient became very restless and had regular labor pains. I was immediately sent for, but being all night engaged with two other obstetric cases, was at the time unable to obey the summons. On my arrival, about 9 o'clock, A. M., I found Dr. Lamb had been called in, who, having understood the nature of the case, acted cautiously, merely relaxing the bandage without removing the tampon. Finding the hemorrhage inconsiderable, I tranquillized the minds of the patient and her friends, thereby rendering important assistance: resigning the case into my hands, Dr. L. withdrew, offering any aid in his power should it be requisite. Labor advanced, *cum pari passu*, and with the most intense anxiety I watched the wonderful operations of nature at this important moment. About noon the expulsive efforts were so strong as to extrude the tampon with a moderate quantity of coagula. This having taken place, and no flooding existing, I found the placenta, as expected, occupying the os uteri, and protruding into the vagina, dilatation of the os uteri being complete. I now sent for Dr. Lamb to witness the case and render assistance if necessary. He came immediately, and found matters as above stated. I requested him to take his seat by the patient and apply his hand over the uterus ready to make firm pressure if such should be requisite. I introduced my hand per vaginam for the twofold purpose of diagnosis and prompt action, and passing my fingers very gently over the edge of the placenta, I found the membranes entire and strong—the head of the child at the superior strait towards the os pubis, with an arm protruding, and the inferior extremities upon the sacrum, the left side of the child presenting. Having made this exploration, I resolved not to interfere until absolutely necessary, but firmly retain my *'vantage ground.* The pains were now strongly expulsive, but the manner of the child's position prevented further descent. I now resolved to rupture the membranes and *turn*; the plan was seconded by my colleague, and speedily effected, by first carrying up the arm over the head and by bringing down the inferior extremities, in doing so I experienced no little difficulty as the limbs were much contorted and wedged down at the superior strait by the uterine contractions. However, without flooding, accident, or other unpleasant circumstance, the delivery was safely completed, to the great satisfaction of all parties. The patient's strength soon rallied, and she has been daily improving, although she has since had an attack of intermittent fever.

*Frankford, Nov. 13th,* 1849.

